# Periscope

**Published:** 1889-12

**Authors:** 


### PERISCOPE.

---

### MEDICINE.

**Weil's Disease.**—After an historical account of this malady, described by Weil and named after him, the author gives a *resumé* of the symptoms of the disease. These are: sudden onset, with pain in the head, fever, a temperature of 102.2°—104° F., and pains in the muscles, especially those of the back. There is great prostration, with active delirium in some cases. Appetite is lost; there is nausea and vomiting, and sometimes diarrhœa. Jaundice soon appears, and may be intense. Albuminuria is almost a constant feature, and there are often casts in the urine. The liver and spleen become augmented in volume; very often there are colicky pains, either limited to the hepatic region or spread over the abdomen generally. The condition of the patient seems serious, when a remission occurs with a fall of temperature, but without, in most cases, much amelioration of the general symptoms. The patient appears to be convalescent, but in many cases a relapse occurs after some days. The temperature rises, but does not attain its former height; the other symptoms may or may not become accentuated. After a relapse occupying some days, the temperature definitely falls, and convalescence begins. The patient is always much enfeebled and wasted, and regains his health slowly. Other occasional symptoms are: erythematous and herpetic eruptions, or rarely rose-red spots; still more rarely, epistaxis and other hæmorrhages. Prodromata are absent; recovery is the rule.

The author considers that under the name of Weil's disease a number of pathological states have been described; he thinks that most of the cases are identical with mild forms of acute yellow atrophy, which is now known to vary greatly in severity in different instances. A smaller number of cases present exactly the same symptoms that Guesinger described under the name "fièvre typhoïde bilieuse"—that is to say, remittent fever complicated with jaundice. Several authors have suggested that Weil's disease is enteric fever complicated with jaundice; but this is improbable, since the symptoms do not correspond, and jaundice, while very rare in typhoid fever, is of constant occurrence in Weil's disease; in the former it is generally followed by death. — Paul Cheron, *L'Union médicale*, May 30th, 1889. J. M. C.

**Tissue-Metabolism and Coma in Cancer.**—The supervention of coma in patients suffering from carcinoma was first noticed by von Jaksch in a case in which the urine contained acetone. Riess and Senator likewise met with it in anæmia and cancer; while lately Oppenheim recorded an instance of coma with convulsions coming on in the course of cancer without any lesion being found in the brain. Anæmia, cancer, and diabetes may thus all end in coma. These are looked upon as cachectic diseases in which the tissue changes are abnormal. The health of each individual depends to a certain extent on the fact that the excretion of urea does not exceed the ingestion of albumen. The nitrogenous equilibrium is an index that the tissue-change is normal. On the other hand, Klemperer, as the result of careful research, has found that an organism afflicted with cancer loses considerably in albuminoid matter even under conditions which, compared with the normal, afford an increase in the quantity of albumen ingested. The food was carefully measured and weighed, and the N. factor calculated, while the fæces and urine were collected and the amount of N. excreted determined by combustion. All the patients in whom the investigations were carried out were free from fever and œdema. Of seven cases of cancer thus estimated by Klemperer, five showed the characteristic increase of albuminoid metabolism. In two cases the overplus of the N. excretion over the ingestion corresponded to those found in insufficiently-nourished individuals. Thus, as an example may be quoted the case of a man with cancer of the stomach who received daily 14.9 grammes of N., and excreted 22.45 gr.—*i.e.* 7.55 grammes N., representing 227 grammes muscle-substance, were lost daily. This shows a destruction of the tissue-albumen. Now this condition is common to all intoxicating processes, *e.g.* phosphorus, arsenic, as well as chloroform poisoning, where there is a tendency for fat deposits to take place in the body and the alkalinity of the blood to be lessened. In all these points cancerous disease agrees closely. From these considerations Klemperer takes the view that a poison circulates in the blood of cancer patients, and that carcinoma represents a poisoning process. This poison causes decomposition of the tissue-albumen, and produces increased N. excretion, internal fat formation, and diminished alkalinity of the blood; while in certain cases it may induce fatal coma. Klemperer gives the histories of two cases which he had observed, and which ended in coma. A parallel may be drawn with diabetes mellitus, which ends not rarely in coma. Here, at least in the serious cases, there is increased metabolism as a rule, while fatty deposits in the internal organs have often been observed.—*Berliner klinische Wochenschrift*, No. 40, 1889. D. R. P.

### THERAPEUTICS.

**Iodide of Potassium in Syphilis.**—M. Fournier begins with a daily amount of 30 grains for an adult man, 22½ for a woman, divided into three doses. He increases this quantity as quickly as possible to 45—60 grains daily, or to 30—45 grains daily for a woman. This last he regards as the average dose which is effectual; it is injurious to give more than 2½—3 drachms daily. Mercury is the true antidote to syphilis; and iodide of potassium is comparatively feeble in its effects, being powerless to prevent tertiary symptoms and fresh outbreaks of the disease. While mercury is the chosen agent for the cure of secondary affections, in some of these iodide is more effectual than mercury; *e.g.* headaches, and painful affections of the bones, joints, and muscles. When tertiary affections come on early, or the general debility of the patient contra-indicates the use of mercury, iodide of potassium is to be given. The iodide is most useful in tertiary manifestations of the disease; but even in these mercury should generally be given as well, to prevent recurrences. This treatment by iodide and mercury combined is especially indicated in cerebral syphilis, in iritis, choroiditis, sarcocele, and deep ulcerations; it is best carried out by giving the drugs separately and not combined in one mixture. The iodide may be given in mixture at meal-times three times a day, with mercurial inunction at night.—FOURNIER, *Gazette des hôpitaux*, March 7th, April 16th, 1889.
J. M. C.

**Removal of Vaginal Cystocele during Hypnotic Sleep.**—The patient was a young woman of 25, who had a large painful vaginal cystocele, which had come on after confinement two years previously. She seemed in good health, and not particularly nervous; but one day the house-surgeon noticed that she looked at him with a peculiar fixed stare, her limbs trembled a little, and then to his astonishment she suddenly fell asleep. It was then ascertained that during the last six years she had fallen asleep whenever she looked fixedly at any object; that her friends could throw her into an hypnotic sleep with great ease; and that M. Bernheim, of Nancy, had studied her case for some time. During the sleep all forms of sensibility were completely abolished, and the tumour could be examined without her feeling it. She appeared to be separated completely from the external world, with the exception of her communications with the hypnotiser; and when she awoke, she knew nothing of what had passed.

M. Tillaux decided to perform colporrhaphy, without chloroform, while she was in this state of hypnotic sleep: the operation lasted twenty minutes, and she showed no sign of suffering pain during its performance. When put back to bed, aroused from sleep, and told that the operation had been done, she brusquely replied that that was not true.—*Gazette des hôpitaux*, August 1st, 1889. J. M. C.

**New Hypnotics.**—The recognition by pharmacologists of the fact that physiological action depends largely on chemical constitution is resulting in the introduction of new remedies, each of which claims in its turn the designation of being a safe hypnotic. Most of them retain the hydrate of chloral as the sleep-producing factor, while at the same time there is an attempt made to overcome its injurious influence on the circulation by the addition of substances which are known to stimulate the vascular centres. Langgaard, in discussing their relative value, says that the greatest interest attaches to chloralamide, a combination of chloral hydrate with formamide. Observations show it to be a useful, although not in all cases an efficient, hypnotic, with few disagreeable after-effects, and especially wanting in influence upon the circulation. The sleep-producing power is less than that of chloral; thus 30 grains of the latter may be considered equal to 45 of the former. Its dose runs from 30—45 grains. Sleep ensues in half an hour to three hours after administration, and its duration varies from 2—9 hours. Its efficacy is most marked in simple nervous sleeplessness; it is useful in delirium tremens, and in the insomnia of heart-disease and of old age. In intense pain it is useless. Its after-effects are relatively few, limited to lassitude and drowsiness on the following day, with perhaps headache and giddiness on awakening. Most observers believe the drug has no action on the respiration and circulation. Langgaard, however, does not agree with this. The frequency of the breathing may not be affected, but the depth is very much diminished. The blood-pressure is lowered, but this is more gradually and more slowly effected than in the case of chloral.

Care is, however, required in the use of the drug in cardiac disease, if one is wishful to avoid unpleasant consequences.

Chloralammonium is a combination of chloral and ammonia. It was tried by Nesbitt in forty cases, in doses of 5—20 grains. (*Therapeutic Gazette*, p. 88, 1888.) He says nothing, however, about its hypnotic power. It is very unstable, dissolves in cold water with decomposition, and has an odour of ammonia and chloroform. Its action has no special advantage. In rabbits the hypnotic effect took place, but along with a considerable sinking of the blood-pressure, even with doses which produced no more than a couple of hours' sleep.

Chloralurethane, according to Poppi, acts quicker and with more certainty than any other, and is free from every after-effect, while it lowers the blood-pressure only in lethal doses. Of this drug Langgaard remarks that it is not easy to see how, as in this compound, the depressing influence of one substance upon the circulation can be counteracted by another which has no stimulating action upon that system. It is almost insoluble in cold water, and is split up into chloral and urethane in boiling. Experiments show that its hypnotic action is not strong, the reflexes being present all the time, and the blood-pressure falling within ten minutes very considerably.

With regard to somnal, which Radlauer has introduced as ethylated chloralurethane and as a perfect hypnotic, it appears to be a solution of a product resulting from the action of urethane upon chloral alcoholate in alcohol. It is not a solid, as represented; it has a more disagreeable taste than chloral, while its effect is less and of shorter duration. Langgaard found the respiration and circulation much influenced. In twenty trials the results were slight and greatly inferior to chloral.—*Therapeutische Monatshefte*, Hefte 10 and 11, 1889. D. R. P.

**Notes on the Choice of Anæsthetics, and on Position and Methods of Anæsthetisation, in Naso-pharyngeal Operations.**—J. F. W. Silk thus summarises the conclusions on the choice of anæsthetics to which his own experience has led him:

1. That the possibility of using nitrous oxide should always be considered, in naso-pharyngeal operations of short duration.

2. Failing this, the advantages and disadvantages of ether, and, by preference, of the combined method of chloroform and ether, should be carefully weighed before deciding the question.

3. That chloroform has drawbacks special to these particular operations; and when employed, its use should, especially in prolonged operations, be limited to the primary induction of narcosis, substituting ether at as early a stage as possible.

As regards the position of the patient:

(*a*) *Supine* has its objections in that the velum palati falls and causes nasal obstruction; the tongue readily slips back, and blood accumulates in the upper part of the pharynx and larynx: the last objection is met by allowing the patient's head to fall over the end of the couch.

Over-anæsthetisation is the chief danger in these operations; therefore ether is preferable to chloroform.

(*b*) *Prone.* All the objections of the supine position are met; but the compression of the chest and prevention of elimination of the anæsthetic is a serious danger, and it is most difficult to keep up anæsthesia with a vapour heavier than air.

(*c*) *Sitting.* For slight operations, *e.g.* tonsillotomy, cauterisation, &c., this is good; its disadvantages being the tendency of the patient, if deeply under, to slip out of the chair, and the difficulty of wiping out the throat if hæmorrhage renders this necessary. The chair ought to be high-backed, with arms, and velvet-covered to prevent slipping; and the head should be pressed forward, to allow blood to escape by the mouth. For scraping adenoid growths, the body should be bent forward, head over a basin, and the blood allowed to flow away by the side of the operator's finger.

As regards methods of administration:

1. *Nitrous oxide.* Care ought to be taken, especially in the case of children, not to injure the gums, soft palate, &c., by using too large a gag. Ether may be combined with it by means of a small Clover's ether-chamber, placed between the face-piece and gas-bag.

2. *Ether* may be administered erect if the narcosis is not required to be very deep, when the patient ought to be supine. It may be kept up either by means of applications of the face-piece or of a gum elastic catheter, attached to a Junker's inhaler and passed into the mouth, the bottle of the inhaler being filled with ether. It may also be used to *maintain*, but not to produce, narcosis by means of a Skinner's cage.

3. *Chloroform.* Administration of this drug in any but the supine position is quite inadmissible. The covering of the

Skinner's cage should be of coarse flannel or domette, and not of ordinary flannel, in order that air may be freely mixed with the drug.

4. *Mixtures*. The rule is, that any mixture containing the slightest trace of chloroform should be administered in the same way and with the same precautions as that agent.

G. Stoker, in opening a discussion on this subject at the British Laryngological Association, considers that of *local* anæsthetics, cocaine is the only one that can be considered. It ought to be used of 20 per cent. strength, freshly made, applied with spray, brush, or plug of cotton-wool soaked in it. Five minutes ought to elapse before anæsthesia is perfect, and it passes off in 10 to 15 minutes. No poisoning results from the drug unless hypodermically injected. It is not advisable in children, as it does not diminish dread of operation. It is not of much use if there are several nasal polypi to be removed, but is good if only a few are removed.

Nitrous oxide is good for short operations, *e.g.* removal of tonsils. This anæsthetic to produce primary anæsthesia, then ether, and lastly chloroform, pumped in by means of Junker's apparatus, are very useful in operations on the air-passages.

The position to be preferred is as follows: The patient lies on his back, with the head and shoulders supported by a thick triangular cushion, the head being on the thickest part of it. Just before the operation begins the patient is drawn up on the cushion, and the head and neck allowed to drop over until the top of the head rests on the couch. The cavity of the nose thus becomes the most dependent part of the air-passages, and into it the blood gravitates, and can neither pass up the food- or air-passages.

Dundas Grant agreed with Mr. Stoker as regards the value of throwing the head well back.

Lennox Browne is not very favourable to the use of general anæsthetics in these operations; but recommends first the use of nitrous oxide, then a little ether, and then resuming nitrous oxide, and thus gets from seventeen to twenty seconds of complete anæsthesia. He warns us against allowing patients to use the cocaine spray, especially in diseases of the nose, as they are apt to induce a continual state of paralysis of the nerves, and thus lead to a condition of chronic anæmia of the parts.

Mr. Orwin likes the A. C. E. mixture, and he operates in the upright position, with the patient so tilted forward that the blood flows out of the mouth.—*Journal of Laryngology and Rhinology*, May and June, 1889. B. J. B.

